# Facile Synthesis of Carbon- and Nitrogen-Doped Iron Borate as a Highly Efficient Single-Component Heterogeneous Photo-Fenton Catalyst under Simulated Solar Irradiation

**DOI:** 10.3390/nano11112853

**Published:** 2021-10-26

**Authors:** Shan-Yuan Hsiao, En-Xuan Lin, Pei-Yuin Keng

**Affiliations:** Department of Materials Science and Engineering, National Tsing Hua University, Hsinchu 300, Taiwan; edwardhsiao16888@gmail.com (S.-Y.H.); a5483892@gmail.com (E.-X.L.)

**Keywords:** photo-Fenton reaction, iron borate, dye degradation, tetracycline, Rhodamine B, azo dyes, metal borates, photocatalysts, dopant

## Abstract

The development of a heterogeneous catalyst for use in environmental remediation remains a challenging and attractive research endeavor. Specifically, for Fenton reactions, most research approaches have focused on the preparation of iron-containing heterostructures as photo-Fenton catalysts that utilize visible light for enhancing the degradation efficiency. Herein, the synthesis and novel application of C,N-doped iron borates are demonstrated as single-component heterogeneous photo-Fenton catalysts with high Fenton activity under visible light. Under the optimal conditions, 10 mg of the catalyst is shown to achieve effective degradation of 10 ppm methylene blue (MB) dye, Rhodamine B (RhB) dye, and tetracycline (TC) under simulated solar irradiation with a first-order rate constant of k = 0.218 min^−1^, 0.177 min^−1^, and 0.116 min^−1^, respectively. Using MB as a model system, the C,N-doped iron borate exhibits 10- and 26-fold increases in catalytic activity relative to that of the 50 nm hematite nanoparticles and that of the non-doped iron borate, respectively, in the presence of H_2_O_2_ under the simulated solar irradiation. Furthermore, the optimum reaction conditions used only 320 equivalents of H_2_O_2_ with respect to the concentration of dye, rather than the several thousand equivalents of H_2_O_2_ used in conventional heterogeneous Fenton catalysts. In addition, the as-prepared C,N-doped iron borate achieves 75% MB degradation after 20 min in the dark, thus enabling the continuous degradation of pollutants at night and in areas with poor light exposure. The stability and recyclability of C,N-doped iron borate for the oxidation of MB was demonstrated over three cycles with insignificant loss in photo-Fenton activity. The high Fenton activity of the C,N-doped iron borate is considered to be due to the synergistic action between the negatively-charged borate ligands and the metal center in promoting the Fenton reaction. Moreover, carbon and nitrogen doping are shown to be critical in modifying the electronic structure and increasing the conductivity of the catalyst. In view of its synthetic simplicity, high efficiency, low cost of reagents, and minimal cost of operation (driven by natural sunlight), the as-prepared heterogeneous single-component metal borate catalyst has potential application in the industrial treatment of wastewater.

## 1. Introduction

The development of a facile, effective, low-cost, stable, and non-toxic water treatment process is becoming a critical issue in the 21st century due to the drastic increases in population, urbanization, and industrialization. Modern industrialization produces by-products and pollutants that have adverse effects upon human health, the environment, and aquatic life. Hence, many innovative methodologies have been applied to the removal of contaminants from waste water, including coagulation, precipitation, electrolysis, sedimentation, ion exchange, and adsorption [1]. However, these physical separation methods fail to completely degrade the pollutants and, thus, produce secondary waste products that are discharged into the environment. Advanced oxidation processes (AOPs) are regarded as highly effective methods for degrading most wastewater pollutants into smaller, non-toxic compounds such as carbon dioxide [2,3]. The AOPs rely on the generation of reactive oxygen species (ROS) in the presence of oxidizing species such as H_2_O_2_ and peroxymonosulfate (PMS). However, the conventional AOP process utilizes oxidizing reagents in large excess (~1000×) with respect to the concentration of pollutants, thus increasing the overall cost of water treatment [4].

Among the various AOP processes, the Fenton reaction has received the most intensive research attention due to its rapid reaction rate, high mineralization efficiency, low cost of operation, easy-to-handle reagents, and the use of non-toxic and earth abundant iron oxides to activate H_2_O_2_. Recent advances include the introduction of electrons to the heterogeneous Fenton catalyst by combination with semiconducting, electron-rich, or plasmonic materials in order to accelerate the rate-limiting step (i.e., the reduction of Fe(III) to the redox active Fe(II) ion) in the decomposition of H_2_O_2_ [5]. Other physical-assisted strategies such as the introduction of an electric field [6], UV/visible light [7,8], ultrasound, and microwave radiation [9,10] have also shown an increase in the generation of hydroxyl radicals, and therefore exhibited a high degradation efficiency under external fields. Notably, the combination of iron oxides with a wide bandgap semiconductor such as TiO_2_ and ZnO has provided heterogeneous photo-Fenton catalysts with significant improvements over the use of iron oxides alone because the photogenerated electrons facilitate the conversion of Fe(III) to Fe(II) [11,12]. Moreover, the highly positive valence bands of TiO_2_ and ZnO provide highly oxidative photogenerated holes that can also participate in the degradation of organic pollutants [13,14]. However, the large bandgaps of these semiconductors limit them to the use of UV light, thus increasing the cost of operation. Hence, researchers have combined iron-containing materials with lower bandgap semiconductors such as CdSe, C_3_N_4_, and BiVO_4_ in order to generate photocarriers under visible light irradiation and, thus, take advantage of the freely-available and unlimited solar energy [15,16,17]. However, while this is an elegant approach, the large-scale implementation of these iron-containing heterojunctions and heterostructures is hampered by the complicated multi-step chemistry required to synthesize them. Therefore, the development of single component Fenton catalysts that possess a large absorbance in the visible light region and simultaneously exhibit high activity toward H_2_O_2_ activation remains an important research endeavor. However, the design of an effective Fenton catalyst requires several key considerations, including the cost of the materials and the cost of operation, along with the degradation efficiency, toxicity, and stability of the catalyst. Various iron-based nanomaterials such as iron oxide, sulfide, carbide, and composite materials have also been explored as potential strategies for enhancing Fenton catalysis [5]. However, to the best of the present authors’ knowledge, iron borate (FeBO_3_) has never been investigated as a photo-Fenton catalyst.

Iron borate is a rhombohedral calcite-type crystal with interesting magnetic, magneto-optical, magneto-acoustic, and resonance characteristics [18,19,20]. There has been renewed interest in the family of metal borates due to their peculiar performance in electrocatalytic water-splitting reactions [21,22]. While the exact mechanism of these reactions remains controversial, it is generally thought to be based on the sacrificial role of the borate ligand in donating electrons to the metal center to provide a more favorable electrochemical route. In Fenton catalysis, an electron-rich metal center also facilitates the conversion of Fe(III) to the redox-active Fe(II) species, thus enhancing H_2_O_2_ decomposition. However, to the best of the present authors’ knowledge, the metal borates have not been explored previously as photo-Fenton catalysts for environmental remediation. Hence, based on the promising approach of modulating the electronic structures, conductivities, and active sites of various redox-based catalysts via doping with small heteroatoms such as carbon and nitrogen [23,24,25,26,27], a novel C,N-doped FeBO_3_ is presented herein as an excellent photo-Fenton catalyst for activating H_2_O_2_ under visible light irradiation. In practical application, the as-prepared catalyst exhibits pseudo first-order kinetics with an apparent rate of 0.218 min^−1^ for the degradation of methylene blue (MB) using only 320 equivalent excess of H_2_O_2_. Compared to the non-doped FeBO_3_, the C,N-doped FeBO_3_ exhibits a 26-fold increase in MB dye degradation efficiency. It is considered that the present results could open up research opportunities into the family of iron borates for Fenton reactions.

## 2. Materials and Methods

### 2.1. Preparation of the C,N-Doped Iron Borate

The C,N-doped iron borate was prepared via a low-temperature annealing method. Briefly, boric acid (3.138 g, 0.050 mol), guanidine hydrochloride (1.617 g, 0.017 mol), and hexamethylenetetramine (0.2375 g, 0.002 mol) were dissolved in distilled water (63 mL) at room temperature. The precursor mixture (12.6 mL) and various amounts of 50-nm Fe_3_O_4_ nanoparticles (50, 100, and 150 mg) were then added to a crucible and sonicated for 30 min at room temperature. Subsequently, the mixture was heated and kept at 80 °C in an oven for 24 h to yield a completely dry solid. The dried solid was then annealed in a furnace at 800 °C under ambient atmospheric pressure. The heating rate was 5 °C min^−1^, and the maximum temperature was maintained for 12 h. The sample was retrieved from the furnace upon cooling down to 90 °C under atmospheric conditions. Finally, the greenish solid was ground into a powder and stored under ambient conditions. The synthesis of C,N-doped FeBO_3_ was repeated for more than 10 times and a typical yield was between determined to be 80–90 mg. The obtained C,N-doped iron borate samples with various amounts of Fe_3_O_4_ nanoparticles are denoted hereafter as the 5 wt.%, 10 wt.%, and 15 wt.% C,N-doped iron borate, respectively.

### 2.2. Characterization

The particle size, morphology, and chemical composition of the C,N-doped iron borate samples were analyzed using a transmission electron microscope (Jeol, JEMARM200FTH, Tokyo, Japan) equipped with energy dispersive X-ray (EDS) mapping. The X-ray diffraction (XRD) patterns were obtained using a Bruker D2 spectrometer, and the photoluminescence emission spectra were obtained using a Perkin Elmer LS55 spectrometer. The optical absorption measurements were performed via UV-visible diffuse reflectance spectroscopy (UV-DRS; Hitachi, U-3900, Tokyo, Japan). In addition, high-resolution X-ray photoelectron spectroscopy (XPS; ULVAC-PHI, PHI Quantera II, Kanagawa, Japan) was performed using Al Kα irradiation. For this procedure, the as-prepared powder was centrifuged twice with DI water and ethanol, then drop-coated onto a silicon substrate. The binding energy was calibrated to an adventitious carbon peak at 284.6 eV. The XPS peak deconvolution and fitting were performed using the CASA XPS software. In addition, ultraviolet photoelectron spectroscopy (UPS) analysis was performed using a ULVAC-PHI PHI 5000 Versaprobe II with He I (21.22 eV) as a photon source with a 5 V bias. The bonding characteristics of the C,N-doped FeBO_3_ samples were examined via Fourier-transform infrared (FTIR) spectroscopy (Bruker Vertex 80v, Billerica, MA, USA). The iron concentrations were measured using an inductively coupled plasma optical emission spectrometer (ICP-OES; Agilent 725, Santa Clara, CL, USA).

### 2.3. Photocatalytic Degradation Experiments 

The photo-Fenton reaction of the as prepared C,N-doped FeBO_3_ was evaluated by the degradation of methylene blue (MB), Rhodamine B (RhB), and tetracycline (TC) under a 150 W solar simulator. MB was used as a model contaminant for optimizing the performance of the C,N-doped iron borate as a photo-Fenton catalyst. In a typical experiment, the C,N-doped FeBO_3_ catalyst (10 mg) was suspended in MB solution (40 mL, 10 ppm), sonicated for 5 min, then stirred for 30 min to achieve adsorption/desorption equilibrium in the dark. This was followed by the addition of 10 mM H_2_O_2_ (40 μL, 4 × 10^–4^ mol) and irradiation under a 150 W solar simulator. During this experiment, 2 mL kinetic samples were extracted from the solution every 5 min until the total reaction time reached 20 min. Each sample was centrifuged to remove any residual catalysts from the solution, and the supernatant was collected to determine the concentration of MB using a UV–vis absorbance spectrophotometer (HITACHI, U-3900) at their maximum absorbance of 663 nm. The reaction rate was evaluated using a pseudo-first-order kinetics model according to the Langmuir-Hinshelwood formula. The cycling stability of the C,N-doped FeBO_3_ was evaluated by using the same catalyst sample for three consecutive runs of dye degradation. After each run, the C,N-doped FeBO_3_ was collected, sonicated with distilled water and ethanol for 10 min, and dried for use in the subsequent reactions. The amount of MB and hydrogen peroxides in the subsequent runs were adjusted according to the amount of catalyst collected after each run. More detailed protocol is available in the Supplementary Materials.

### 2.4. Active Species Analysis 

The main reactive oxygen species (ROS) that were responsible for the MB degradation using the C,N-doped FeBO_3_ were evaluated by adding various trapping reagents, namely isopropanol (IPA, 6.1 μL, 1 mM), ethylenediaminetetraacetic acid disodium salt dihydrate (EDTA-2Na, 26.9 mg, 1 mM), and 2,2,6,6-tetramethylpiperidine-1-oxyl (TEMPO, 12.2 mg, 1 mM) to scavenge ·OH, h^+^, and ·O_2_-, into the MB degradation experiments. The formation of the hydroxyl radical was confirmed and quantified by adding an excess amount of terephthalic acid (TA; 40 mL, 3.3 mg, 5 × 10^−4^ M) and measuring the production of 2-hydroxy terephthalic acid (2-HTA) according to its intense photoluminescence at 430 nm upon photoexcitation at 332 nm.

## 3. Results

**Figure 1:**
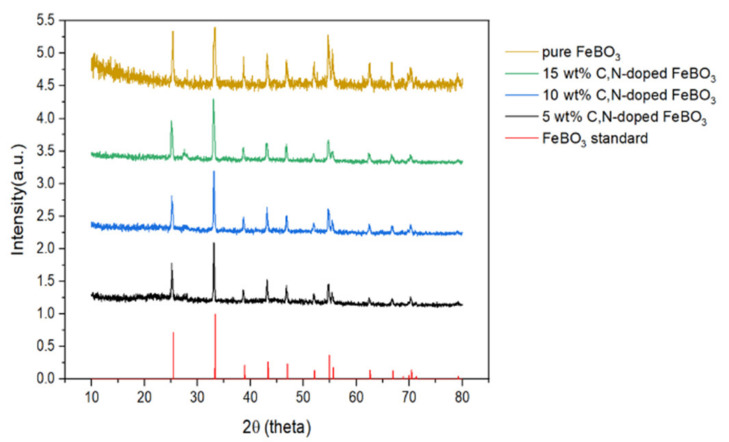
The XRD patterns of the various as-prepared C,N-doped FeBO_3_ samples.

The XRD patterns of the as-prepared C,N-doped FeBO_3_ samples with various quantities of iron oxide nanoparticles are presented in Figure 1, and are all well matched to that of pure FeBO_3_ (JCPDS No. 76-0701). Specifically, FeBO_3_ adopts a calcite crystalline structure that belongs to the rhombohedral space group R3 ®C. The XRD spectra contain no diffraction peaks corresponding to Fe_3_O_4_ or boron carbon oxynitride (BCNO) [28,29], thus indicating that all the Fe and B species were converted into the C,N-doped FeBO_3_ nanocomposite. Moreover, since the XRD patterns of the C,N-doped FeBO_3_ do not contain any other impurity peaks and do not exhibit peak broadening or peak shifting, the results demonstrate that the carbon and nitrogen atoms were fully incorporated into the interstitial spacing of iron borate crystal structure. 

**Figure 2:**
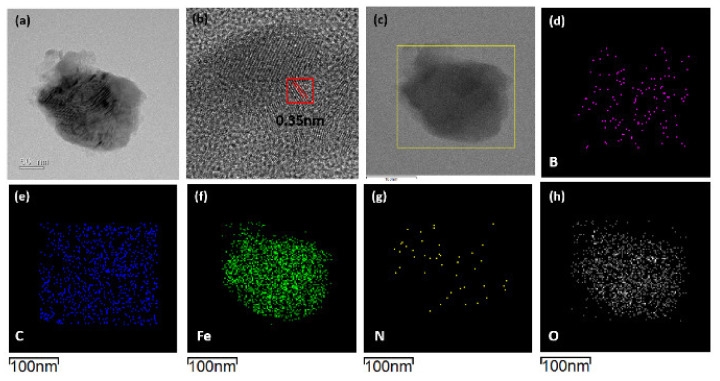
The morphology and chemical composition of the as-prepared iron borate: (**a**,**b**) low-resolution (**a**) and high-resolution (**b**) TEM images; (**c**) STEM image, and (**d**–**h**) corresponding EDX mapping images for the elements B, C, Fe, N, and O. EDX mapping images were taken within the yellow square in Figure 2c.

The HR-TEM, STEM, and EDX mapping images of the C,N-doped FeBO_3_ are presented in Figure 2. Here, the as-prepared C,N-doped FeBO_3_ particle is seen to be irregular in shape, with an average size of 197$\;\pm \;32\;nm\;\left(n=13\right)$. Additionally, the HR-TEM image suggests that the particles are polycrystalline in nature, with a random lattice orientation and an interplanar fringe spacing of 0.35 nm (Figure 2b, red square), which corresponds to the (012) lattice plane of FeBO_3_. The elemental mappings in Figure 2d–h indicate that the elements B, C, Fe, N, and O are homogeneously distributed throughout the irregularly-shaped particle, thus confirming the successful doping of the as-prepared FeBO_3_ with carbon and nitrogen.

**Figure 3:**
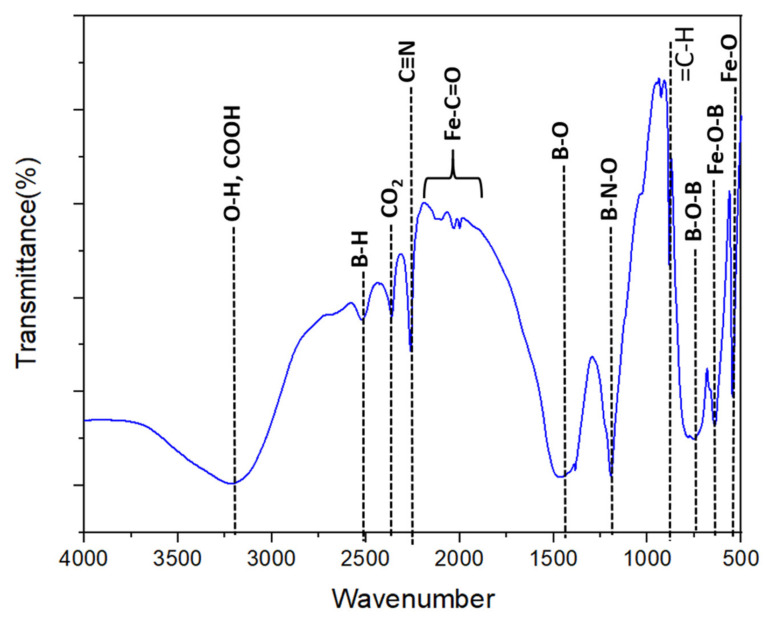
The FTIR spectrum of the as-prepared C,N-doped FeBO_3_.

The FTIR spectrum of the as-prepared C,N-doped FeBO_3_ is presented in Figure 3. Here, the characteristic absorption bands of the borate group are observed, including the B–O stretching vibration at 1384–1460 cm^−1^ and the B–O–B stretching vibration at 749–781 cm^−1^. In addition, the two absorptions at 642 cm^−1^ and 546 cm^−1^ in the fingerprint region correspond to the stretching vibrations of Fe–O and Fe–O–B, respectively [19,30,31,32,33,34]. Further, the broad band at 3200 cm^−1^ due to the O–H and COOH stretching vibration can be explained by the presence of adsorbed atmospheric water and the formation of formic acid in the presence of iron containing compound, respectively. The formation of formic acid could be due to the decomposition of nitrogen precursors to ammonia and formaldehyde during the thermal annealing reaction in the presence of iron oxides [35]. Subsequently, catalytic oxidation of formaldehyde resulted in the formation of formic acid and carbon dioxide. The signature infrared (IR) bands for these compounds are shown in Figure 3, in which the carboxylic acid O–H stretching is embedded in the broad absorption ~ 3200 cm^−1^, while the carbonyl (C=O) band can be visualized as a shoulder around 1650 cm^−1^ wavenumber. Moreover, the physisorption of carbon dioxides onto the metal center can be clearly observed at 2350 cm^−1^ as the asymmetric stretching of CO_2_ [36,37]. The carbon doping into FeBO_3_ can also be visualized through the formation of iron carbonyl (Fe–C=O) bonding between 1900 cm^−1^ and 2100 cm^−1^ (Figure 3). The other distinct IR band at 2500 cm^−1^ is assigned to the hydride vibration of borane (B–H) [38,39]. 

**Figure 4:**
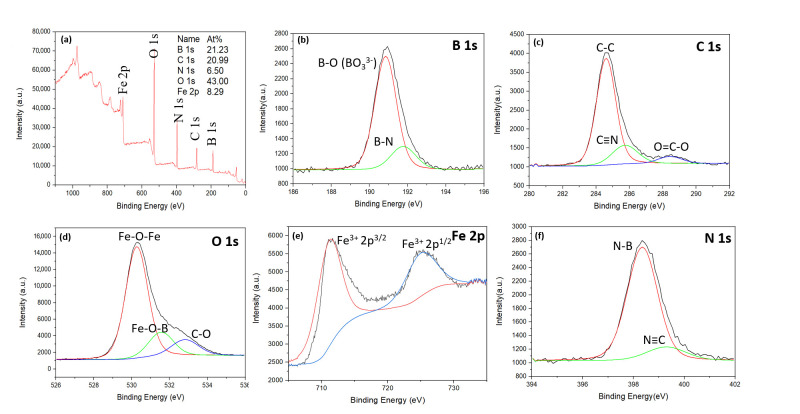
The XPS spectrum of the as-prepared C,N-doped FeBO_3_: (**a**) full survey spectrum, (**b**–**f**) Gaussian fitted B 1s (**b**), C 1s (**c**), O 1s (**d**), Fe 2p (**e**), and N 1s (**f**) spectra.

The XPS analysis of the as-prepared C,N-doped FeBO_3_ is presented in Figure 4. Here, the XPS survey spectrum (Figure 4a) confirms the presence of the elements B, C, N, O, and Fe, with the significant percentage of C and N further confirming the successful doping (Table S1). Moreover, compared to the full XPS spectrum of the pure FeBO_3_ (Figure S1), that of the pure FeBO_3_ XPS exhibits no nitrogen signal. The residual carbon signal in the XPS as shown in Figure S1 can be explained by the adsorption of carbon dioxide during handling under atmospheric conditions.

To gain a deeper insight into the boron, carbon, nitrogen, and iron-related chemical bonding, the Gaussian fitted high-resolution XPS spectra (R^2^ > 0.99) are presented in Figure 4b–f. Thus, the B 1s spectrum in Figure 4b is composed of two fitted curves originating from the B–O bond at a binding energy of 190.8 eV and the B–N–O bonds at 191.9 eV [40,41]. Similarly, the N 1s spectrum (Figure 4f) can be fitted with two Gaussian curves due to the N–B bonding at 398.3 eV and the N–C bonding at 399.2 eV [33,42]. Meanwhile, the C 1s spectrum (Figure 4c) is composed of three peaks at 284.5, 285.7, and 288.1 eV due to the C–C, C–N and C–O–C species, respectively [23,33]. Taken together, the observed B–N–O, N–B, and C–N signals suggest that the C and N dopants are bonded to the borate anion within the FeBO_3_ crystal. Further, the O 1s spectrum (Figure 4d) is deconvoluted into four peaks. The peaks at 530.2 and 531.1 eV correspond to the Fe–O–Fe and Fe–O–B bonding, while that at 532.2 eV corresponds to the C–O bond [22]. The additional weak and broad peak could be due to the adsorbed H_2_O and CO_2_ under atmospheric conditions. Finally, the Fe 2p spectrum reveals two peaks at 710.1 and 724.1eV, which are attributed to the Fe 2p^3/2^ and Fe 2p^1/2^ of FeBO_3_, thus indicating the Fe^3+^ valence state [43].

**Figure 5:**
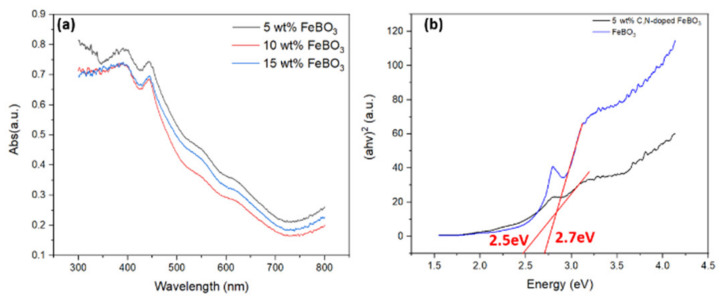
(**a**) The UV-DRS spectra of the C,N-doped FeBO_3_ with various iron contents, and (**b**) the Tauc plots of the C,N-doped FeBO_3_ with 5 wt.% iron nanoparticles (black line) and the pristine FeBO_3_ (blue line).

The UV-DRS results for the heterogeneous C,N-doped FeBO_3_ photo-Fenton catalysts with various weight percentages of iron nanoparticles are presented in Figure 4a. Here, each sample exhibits a broad absorbance in the visible region, and a large absorption band between 400 and 500 nm. The corresponding Tauc plot of the sample with 5 wt.% iron nanoparticles in Figure 5b indicates a bandgap of 2.5 eV, which is slightly lower than that of the pure FeBO_3_ (2.7eV) [44]. This is consistent with the results of carbon and nitrogen doping into the frameworks of various nanomaterials [27] and favors the utilization of most of the solar spectrum by the as-prepared C,N-doped FeBO_3_ catalyst, thus contributing to an enhanced photo-Fenton activity. Further, the valence band edges of the vacuum levels derived from the secondary electron cutoff of the UPS spectra in Figure S2 indicate that the valence band position of the C,N-doped FeBO_3_ is 2.37eV versus the normal hydrogen electrode (NHE).

### Photocatalytic Activity

**Figure 6:**
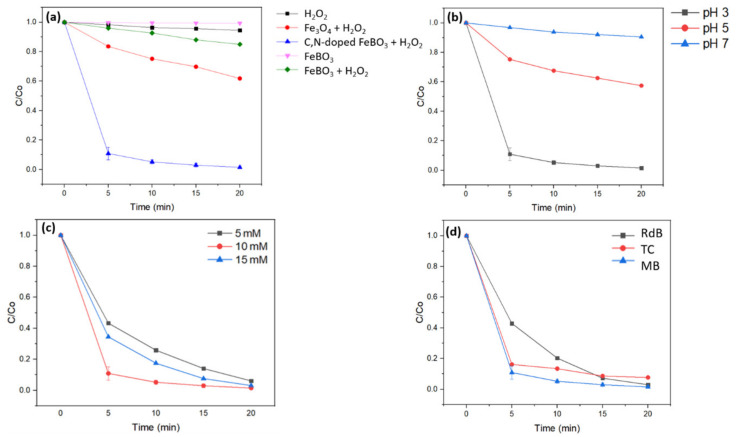
The MB degradation curves under simulated A.M 1.5 solar illumination for (**a**) the 5 wt.% photo-Fenton catalysts in the presence and absence of H_2_O_2_, (**b**) the 5 wt.% C,N-doped FeBO_3_ and H_2_O_2_ at various pH values, (**c**) the 5 wt.% C,N-doped FeBO_3_ and H_2_O_2_ at various H_2_O_2_ concentrations, and (**d**) the photo-Fenton degradation activities for different pollutants using the 5 wt.% C,N-doped iron borate catalyst (pH = 3, H_2_O_2_ concentration = 10 mM, pollutant concentration = 10 ppm).

The photocatalytic room-temperature MB degradation curves of the pristine Fe_3_O_4_ nanoparticles and the 5 wt.% C,N-doped FeBO_3_ particles in the presence and absence of hydrogen peroxide are presented in Figure 6a. Here, the MB does not undergo significant self-decomposition under visible light during the 20 min period in the presence of H_2_O_2_ only (black line). However, in the presence of the pristine 50-nm Fe_3_O_4_ nanoparticles and H_2_O_2_ (red line), only 25% of the MB is degraded within 20 min under the prescribed photo-Fenton reaction conditions. Moreover, the highest catalytic activity is indicated for the 5 wt.% C,N-doped FeBO_3_ in the presence of H_2_O_2_ (blue line), with a degradation rate constant of 0.462 min^−1^ in the first 5 min, and complete MB degradation by 20 min (Figure S3). 

Under the same photo-Fenton conditions, the pure FeBO_3_ synthesized according to literature [45] exhibits only 16% MB degradation after 20 min (green line, Figure 6a, synthesis of pure FeBO_3_ is available in the Supplementary Materials). As a proof of concept demonstration, the photo-Fenton oxidation of other common pollutants such as tetracycline and Rhodamine B using the as prepared 5 wt.% C,N-doped iron borate achieved 92% and 100% degradation efficiency, respectively (Figure 6d). Thus, compared to other iron-based photo-Fenton catalysts, the as-prepared 5 wt.% C,N-doped FeBO_3_ is a superior photo-Fenton catalyst with a potential of removing major organic pollutants with high efficiency during wastewater treatment. These results demonstrate that the carbon and nitrogen doping of FeBO_3_ plays a critical role in creating electrically charged sites, thus explaining the marked increase in the photocatalytic activity of the C,N-doped FeBO_3_ compared to their non-doped counterpart. Moreover, in the absence of H_2_O_2_, the C,N-doped FeBO_3_ particles fail to degrade MB (purple line, Figure 6a), thus indicating that the main catalytic mechanism is the Fenton reaction. 

In summary, the present study has demonstrated that the novel C,N-doped FeBO_3_ catalyst exhibits 10- and 26-fold enhancements in photo-Fenton activity compared to that of the Fe_3_O_4_ nanoparticles alone and that of the non-doped FeBO_3_, respectively, under simulated solar irradiation. Further, the present study has demonstrated that the as-prepared C,N-doped FeBO_3_ catalysts exhibit high pseudo first-order kinetic rates compared to those of other recently-reported catalysts, as summarized in Table S2.

The ICP-OES analyses of the C,N-doped FeBO_3_ samples prepared with various proportions of Fe_3_O_4_ nanoparticles are presented in Table S3, where the percentage of Fe in the final product is seen to match the weight percent of Fe_3_O_4_ nanoparticle added to the precursor mixtures. The MB degradation efficiency of the C,N-doped FeBO_3_ samples with various proportions of iron nanoparticles decreases with increasing Fe content as shown in Figure S4. The iron content of the C,N-doped FeBO_3_ is essential to the photo Fenton system. Generally, a higher iron content represents a larger number of redox active sites for generating ROS for the degradation of pollutants. However, an excessive iron content could also generate recombination centers for the photogenerated holes and electrons, thus removing the photocarriers that would otherwise assist in the photo-Fenton reaction [42].

In view of these results, the most active 5 wt.% C,N-doped FeBO_3_ was used in further investigations to determine the optimal photo-Fenton reaction pH conditions, as shown in Figure 6b. Thus, as with the majority of iron-based Fenton catalysts, the C,N-doped FeBO_3_ exhibits the highest Fenton activity at pH 3. However, the degradation rate of the 5 wt.% C,N-doped iron borate under this optimal condition is superior to that of most other Fenton catalysts, as shown in Table S2. Furthermore, the results in Figure 6 demonstrate that the quantitative degradation of MB dye can be achieved in 20 min in the presence of a minimal amount of H_2_O_2_ (~320 mole equivalent with respect to the concentration of pollutant). In addition, the MB degradation rates of the 5 wt.% C,N-doped iron borate in the presence of various concentrations of H_2_O_2_ (5, 10, and 15 mM) are presented in Figure 6c. Interestingly, a higher concentration of H_2_O_2_ is seen to result in a lower degradation rate during the initial period due to the competitive scavenging effect of the excess H_2_O_2_, as described previously. Finally, the MB degradation rates of the 5 wt.% C,N-doped iron borate under illumination and in the dark are compared in Figure S5. Remarkably, 75% MB degradation is achieved in 20 min even under dark conditions. Thus, it is believed that this proof-of-concept demonstration will facilitate the development of a new class of iron borate materials as efficient Fenton catalysts for water treatment applications both under sunlight and in the dark.

**Figure 7:**
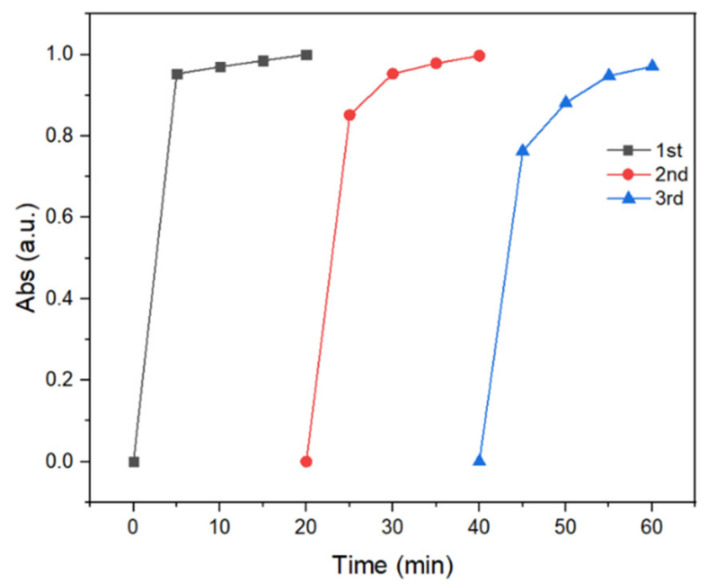
MB dye degradation efficiency of using C,N-doped FeBO_3_ for consecutive three experiments. The mass of catalyst used was 50 mg, 16 mg, and 9 mg, respectively.

The stability and reusability of the as-prepared C,N-doped FeBO_3_ are shown in Figure 7, where the photo-Fenton efficiency remained quantitative after three successive cycling runs. The concentrations of MB and H_2_O_2_ in the subsequent photo-Fenton oxidation reactions were adjusted accordingly to accommodate for the loss of catalyst during the repeated washing and filtration. Moreover, the C,N-doped iron borate exhibits remarkable stability under the prescribed photo-Fenton condition (simulated solar light, pH 3) as evidenced by the low iron concentration in solution after 20 and 60 min of dye degradation reaction (Table S4). Furthermore, the XRD diffraction patterns of the recovered solid catalyst obtained after the photo-Fenton reaction (Figure S6) are seen to be identical to those of the pristine, as-prepared C,N-doped iron borate in Figure 1, thus indicating that the catalyst is stable and can withstand photocorrosion under the simulated solar irradiation.

**Figure 8:**
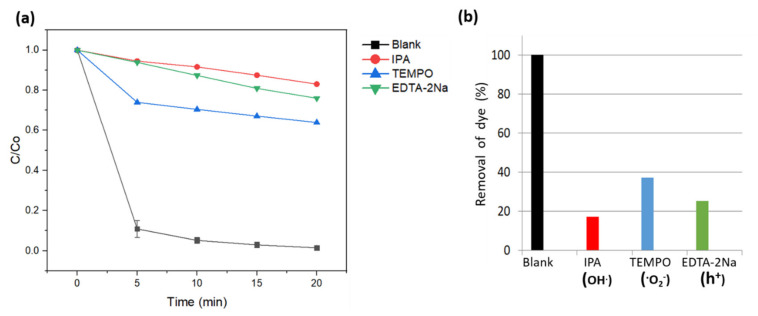
(**a**) The photodegradation of MB by the as-prepared C,N-doped FeBO_3_ under simulated solar illumination in the presence of various scavengers: (**a**) a plot of C/Co; (**b**) a bar graph showing the percentage removal of MB.

The dominant ROS species contributing to the photo-Fenton activity of the C,N-doped FeBO_3_ catalyst are revealed by the selective trapping results in Figure 8. Here, the photo-Fenton activity is seen to be significantly suppressed upon the addition of the ·OH scavenger isopropanol [46] thus indicating that the main ROS responsible for the degradation of MB is the hydroxyl radical. Meanwhile, the h^+^ and^·^O_2_^–^ superoxide species play secondary and tertiary roles, respectively, in the photo-Fenton reaction. Thus, as revealed by the purple line in Figure 6a, the C,N-doped FeBO_3_ shows no photocatalytic activity in the absence of H_2_O_2_. Under photo-Fenton conditions, however, the photogenerated charge carrier is rapidly injected into the Fe^3+^ site to form the redox-active Fe^2+^ while generating ROS via the reaction with H_2_O_2_. The resulting rapid consumption of photogenerated electrons enables the photogenerated holes to also participate in the MB dye degradation.

**Figure 9:**
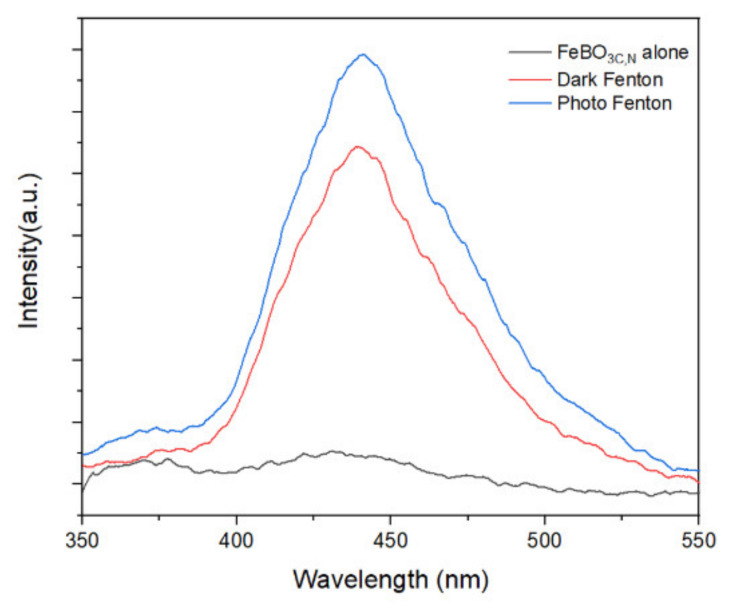
The TAOH fluorescence spectra of the C,N-doped FeBO_3_ alone under the dark-Fenton reaction and photo-Fenton reaction for 20 min.

The role of ·OH as the dominant ROS species in the photo-Fenton system is further verified by the use of terephthalic acid as a hydroxyl radical probe. Thus, in Figure 9, the fluorescence intensity of 2-hydroxyphthatlic acid directly corresponds to the concentration of hydroxyl radicals formed during the Fenton reaction [47]. As anticipated, a much higher concentration of hydroxyl radical is produced under the simulated solar irradiation than in the dark, thus explaining the slightly higher degradation efficiency under photo-Fenton conditions. In the dark, the C,N-doped FeBO_3_ is also capable of producing a significant amount of hydroxyl radical, commensurate with the observed dark-Fenton activity in Figure 9. This result demonstrates that the as-prepared C,N-doped FeBO_3_ will be able to operate continuously with a high degradation efficiency both in the presence and absence of sunlight, thus reducing the overall amount of time needed to treat a particular batch of wastewater. Furthermore, these catalysts open up the possibility of treating waste water in areas that do not receive sufficient sunlight.

**Figure 10:**
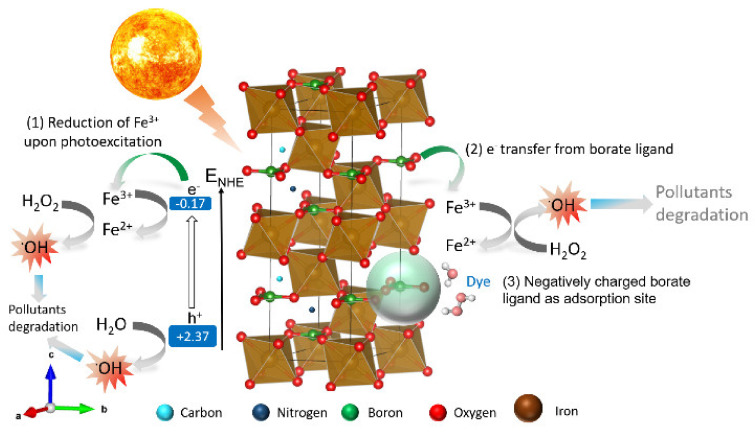
A schematic diagram showing the unit cell of C,N-doped FeBO_3_ and the proposed mechanism of MB degradation on the C,N-doped FeBO_3_ system. The aqua blue spheres—carbon, blue—nitrogen, green—boron, red—oxygen, and brown—iron.

The above-mentioned controlled and trapping experiments demonstrate that the dye degradation mechanism of the C,N-doped FeBO_3_ follows the standard Fenton reaction. As shown schematically in Figure 10, the peculiar activity of the C,N-doped FeBO_3_ catalyst in Fenton reactions might be explained by the synergistic effect of the negatively-charged borate ligands [48,49]. According to in-depth studies of metal borates as electrocatalysts, the borate ligands serve as sacrificial ligands for the formation of a redox-active metal site via a reverse electron transfer mechanism. As a result, the iron center is filled with more electrons and, thus, can readily be reduced to form the redox-active Fe^2+^. In addition, the negatively-charged borate ligand serves as a preferential adsorption site for MB and H_2_O_2_/H_2_O, which consequently shortens the charge transfer distance between the Fe^2+^/Fe^3+^ and H_2_O_2_ to produce the reactive hydroxyl radicals [50]. Under photo-Fenton conditions, a 25% increase in Fenton activity could be attributed to the injection of the photogenerated electrons into the Fe^2+^/Fe^3+^ site. Based on the band gap and band edge analysis of the C,N-doped FeBO_3_, the conduction band is located at −0.17 eV vs. NHE, while the Fe^3+^/Fe^2+^ redox potential is at 0.77 eV. The more negative conduction band of the C,N-doped FeBO_3_ allows the direct injection of the photogenerated electrons into the Fe^3+^/Fe^2+^ cycle to overcome the rate limiting step of the Fenton reaction.

Meanwhile, the photogenerated holes (h^+^) that are formed at the highly positive valence band of the C,N-doped FeBO_3_ could directly oxidize the adsorbed water or hydroxyl anion to produce the reactive radical via the reactions in Equations (1) and (2):

h^+^ + OH^−^ → ^·^OH (1.99 eV vs. NHE) (1)

h^+^ + H_2_O → ·OH + H^+^ (2.27 eV vs. NHE) (2)

Furthermore, the carbon and nitrogen doping of the FeBO_3_ plays a critical role in the 26-fold enhancement in the Fenton activity relative to that of the non-doped FeBO_3_. Carbon, nitrogen, and boron dopants are commonly used for improving the catalytic activity of various catalysts. For example, nitrogen-doped catalysts generally show an increase in the number of catalytic sites along with improved adsorption of active species, thus facilitating the electric conductivity by providing electron carriers and decreasing the band gap energies [51]. As shown in Figure 5b, the bandgap of the C,N-doped FeBO_3_ is smaller than that of the un-doped FeBO_3_, which is consistent with the effect of carbon and nitrogen in modulating the electronic properties of other catalysts. Although further in operando investigations are needed in order to elucidate the mechanism of FeBO_3_ in promoting the Fenton activity, this initial proof-of-concept helps to provide a basic understanding of the utilization of a new class of iron materials as effective (photo)-Fenton catalysts.

## 4. Conclusions

In this study, a novel C,N-doped FeBO_3_ catalyst was synthesized via a simple and cost-effective method. The most optimal C,N-doped FeBO_3_ catalyst was prepared using 5 wt.% Fe_3_O_4_ nanoparticles. The highest photo-Fenton activity was obtained using 10 mM of H_2_O_2_ in 10 ppm concentration of MB dye at pH 3. Moreover, the as prepared catalyst was shown to remove various organic pollutants with high efficiency under simulated solar light irradiation. Based on the band edges of the C,N-doped FeBO_3_, the photogenerated electrons can be directly injected into the Fe^3+^/Fe^2+^ site, thus increasing the Fenton activity upon solar irradiation. The facile Fenton activity of FeBO_3_ could be attributed to the synergistic effects of the borate ligand and the metal center of the FeBO_3_ framework. Furthermore, the carbon and nitrogen dopants effectively modified the electronic structure, facilitated charge transport, and increased the conductivity, thus contributing to the excellent catalytic activity over the non-doped FeBO_3_. This study provides a simple and facile methodology for the preparation of a highly stable and active single component, metal borate-based photo-Fenton catalyst capable of absorbing visible light that could potentially bridge the gap between academic research and industrial application in wastewater remediation.

## Supplementary Materials

The following are available online at https://www.mdpi.com/article/10.3390/nano11112853/s1: Synthesis of the pure iron borate; Figure S1: XPS full spectra with fitting curves of (a) FeBO_3_ and (b) C, N-doped FeBO_3_, Figure S2. UPS spectra of C, N-doped FeBO_3_,Figure S3. Pseudo-first-order rate reaction kinetics for MB dye with different condition; Figure S4: Degradation curves of methylene blue under A.M 1.5 solar simulator using different iron content C,N-doped FeBO_3_ + H_2_O_2_ as photo-Fenton catalyst; Figure S5. The comparative MB photodegradation performance of the 5 wt.% C,N-doped FeBO_3_ in the dark and under illumination; Figure S6. The comparison of XRD pattern before and after photocatalytic experiment. Table S1: XPS elemental composition of the 5 wt. % C,N-doped FeBO_3_; Table S2. Representative works of other photo-Fenton catalysts and their efficiency in the degradation of methylene blue dye at pH 3; Table S3. ICP-OES of the solid C, N-doped FeBO_3_ prepared using different weight percentage of Fe_3_O_4_ nanoparticles; Table S4. Quantitative measurement of iron concentration leached out into the solution after 20.
