# Supplementary material for: Facile Synthesis of Carbon- and Nitrogen-Doped Iron Borate as a Highly Efficient Single-Component Heterogeneous Photo-Fenton Catalyst under Simulated Solar Irradiation
Source: Nanomaterials (Basel). 2021 Oct 26;11(11):2853. doi: 10.3390/nano11112853 (PMC8620105; doi:10.3390/nano11112853)
Supplement: Supplementary file 1 [file nanomaterials-11-02853-s001.zip › nanomaterials-1432080-supplementary.pdf]

Supplementary Materials

Facile Synthesis of Carbon- and Nitrogen-Doped Iron Borate as a Highly Efficient Single-Component Heterogeneous Photo-Fenton Catalyst under Simulated Solar Irradiation

Shan-Yuan Hsiao, En-Xuan Lin and Pei-Yuin Keng *

Department of Materials Science and Engineering, National Tsing Hua University,
Hsinchu 300, Taiwan, China; edwardhsiao16888@gmail.com (S.-Y.H.); a5483892@gmail.com (E.-X.L.)
* Correspondence: keng.py@gapp.nthu.edu.tw

Synthesis of pure iron borate. The $Fe(C_2O_4){\cdot}H_2O$ (1 g, 5.5 mmol) and H_3BO_3 (3 g, 48 mmol) were mixed in a molar ratio of 1:3. Then, the mixture was calcined at 750 °C for 15 h under an air atmosphere. Finally, the $FeBO_3$ was obtained by washing the precursor with boiling deionized water and drying it in an oven at 80 °C.

Table S1: XPS elemental composition of the 5 wt. % C,N-doped $FeBO_3$

Element	B	C	N	O	Fe
Composition	27.7%	17.4%	15.3%	33.3%	6.3%

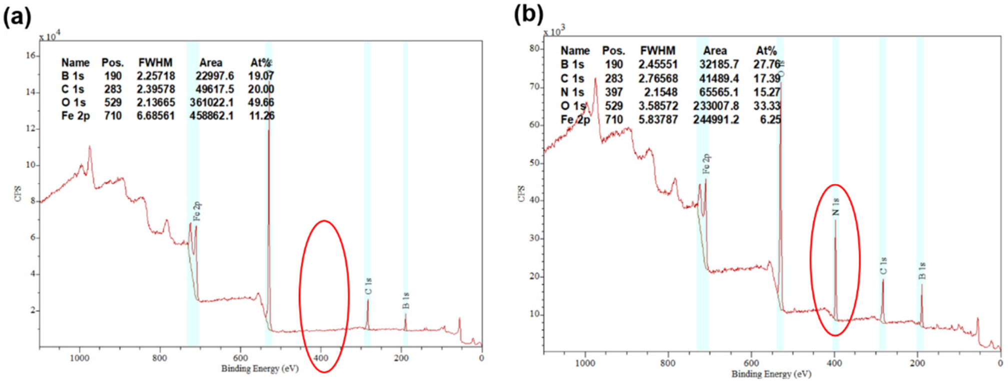

Figure S1. XPS full spectra with fitting curves of (a) $FeBO_3$ and (b) C, N-doped$FeBO_3$

Recyclability of C,N-doped $FeBO_3$. The cycling stability of the C,N-doped $FeBO_3$ was evaluated by using the same catalyst sample for three consecutive runs of dye degradation. After each run, the C,N-doped was collected, sonicated with distilled water and ethanol for 10 min, and dried for use in the subsequent reactions. The amount of MB and hydrogen peroxides in the subsequent runs were adjusted according to the amount of catalyst collected after each run. The first dye degradation cycle was performed by adding 50 mg of the C, N-doped $FeBO_3$ catalyst in 200 mL of MB solution (10 ppm). After sonication for 30 minutes, 10 mM H_2O_2 (200 μL, 2 x 10_{-3} mol) was added into the catalyst suspension. The used catalyst was collected, sonicated with distilled water and ethanol for 10 minutes, and dried for use in the second photo-Fenton reaction. In the second run, 15 mg of the residual C, N-doped $FeBO_3$ was suspended in MB solution (60 mL, 10 ppm). After sonication for 30 minutes, H_2O_2 (60 μL, 6 x 10^{-4} mol, 10 mM) was added into the catalyst suspension. In the third run the residual C,N-doped $FeBO_3$ (10 mg) was suspended in MB solution (40 mL, 10 ppm). After sonication for 30 minutes, H_2O_2 (40 μL, 4 x 10-4 mol, 10 mM) was added into the catalyst suspension.

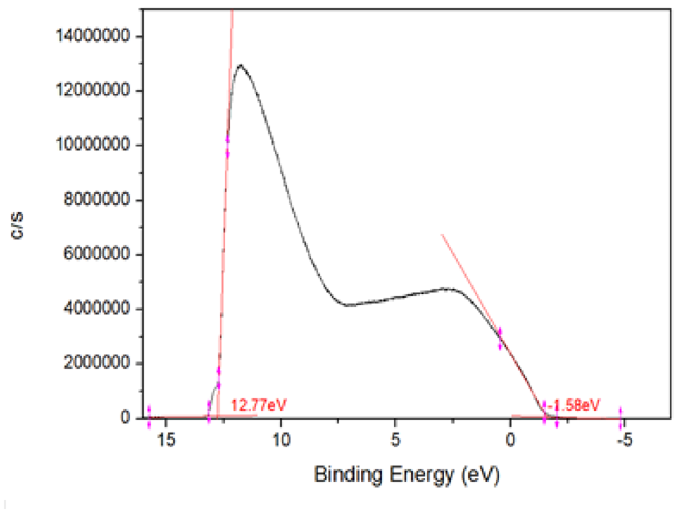

Figure S2. UPS spectra of C, N-doped $FeBO_3$

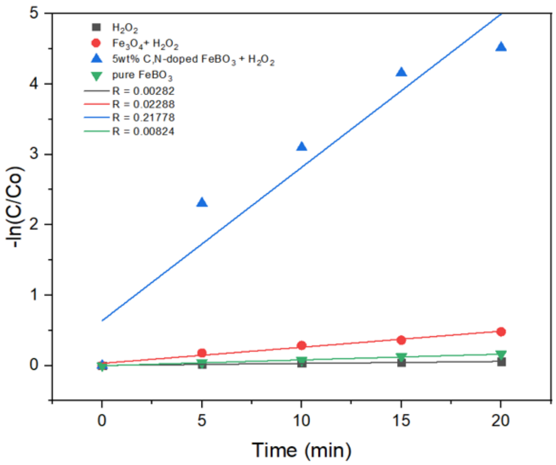

Figure S3. Pseudo-first-order rate reaction kinetics for MB dye with different condition.

Table S2. Representative works of other photo-Fenton catalysts and their efficiency in the degradation of methylene blue dye at pH 3.

**Ref**	**Light source**	**Dye concentration and volume**	**H_2O_2**	**Ratio of H_2O_2/Dye**	**Catalyst loading mg/L**	**Efficiency, *k* *(min^{-1})***
1	350W visible light	MB 5ppm 50ml	128mM	1230	600	0.188
2	350W visible light	Phenol 20ppm 50mL	8mM	38	1000	0.145
3	300W full spectrum	MB 25 ppm, 100 mL	20 mM	256	150	0.063
4	Not mentioned	MB 5 ppm, 50 mL	10 mM	640	2.92	0.058
**This work**	**150 Solar simulators**	**MB 10 ppm, 40 mL**	**10 mM**	**320**	**250**	**0.212**

Table S3. ICP-OES of the solid C, N-doped $FeBO_3$ prepared using different weight percentage of Fe_3O_4 nanoparticles.

	Fe	B
5 wt% C, N-doped $FeBO_3$	5.393	17.41
10 wt% C, N-doped $FeBO_3$	11.17	20.25
15 wt% C, N-doped $FeBO_3$	14.65	18.82

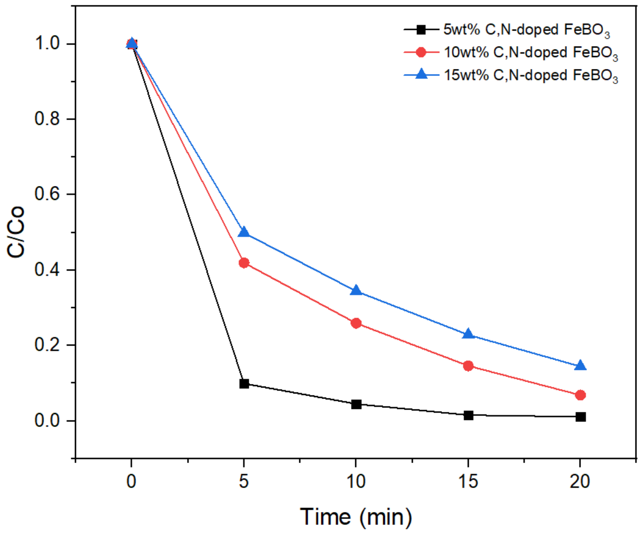

Figure S4: Degradation curves of methylene blue under A.M 1.5 solar simulator using different iron content C,N-doped $FeBO_3$ + H_2O_2 as photo-Fenton catalyst.

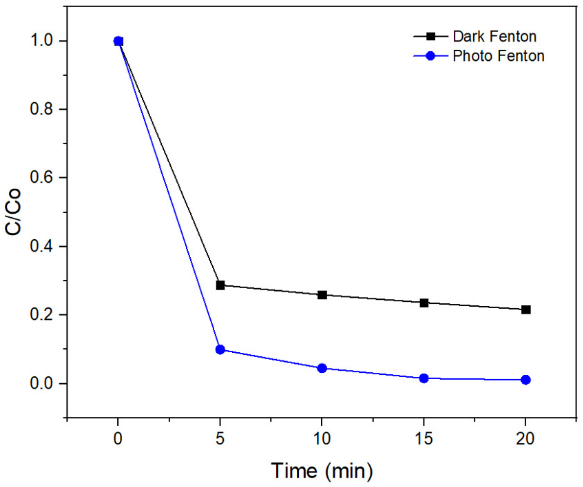

Figure S5. The comparative MB photodegradation performance of the 5 wt.% C,N-doped FeBO3 in the dark and under illumination

Table S4. Quantitative measurement of iron concentration leached out into the solution after 20

	Fe (ppm)
20min	0.478
60min	0.578

minutes and 60 minutes of photo-Fenton reaction (simulated solar light, pH 3).

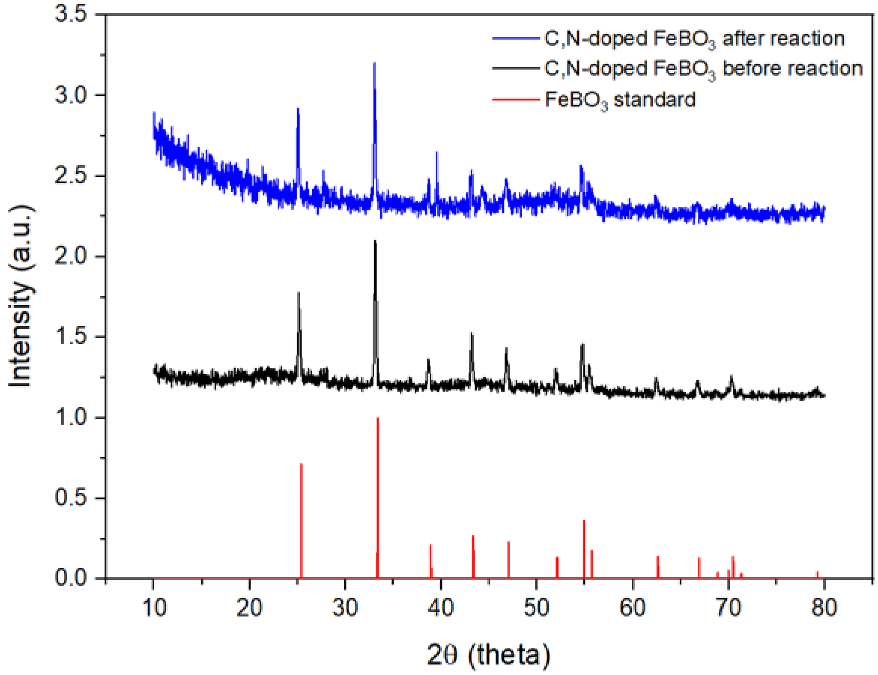

Fig S6. The comparison of XRD pattern before and after photocatalytic experiment

Reference

1. Journal of Water Process Engineering 34 (2020) 101089
2. Applied Catalysis B: Environmental 245 (2019) 130-142
3. Journal of environmental sciences 111 (2022) 11–23
4. ACS Appl. Nano Mater. 2019, 2, 7074−7084
